# Radiation-induced overexpression of transthyretin inhibits retinol-mediated hippocampal neurogenesis

**DOI:** 10.1038/s41598-018-26762-1

**Published:** 2018-05-30

**Authors:** JiHoon Kang, Wanyeon Kim, HyunJeong Seo, EunGi Kim, Beomseok Son, Sungmin Lee, Gaeul Park, Sunmi Jo, Changjong Moon, HyeSook Youn, BuHyun Youn

**Affiliations:** 1 Department of Integrated Biological Science, Pusan National University, Busan, 46241 Republic of Korea; 2 Department of Biological Sciences, Pusan National University, Busan, 46241 Republic of Korea; 3 Department of Radiation Oncology, Haeundae Paik Hospital, Inje University School of Medicine, Busan, 48108 Republic of Korea; 4 Department of Veterinary Anatomy, College of Veterinary Medicine and Animal Medical Institute, Chonnam National University, Gwangju, 61186 Republic of Korea; 5 Department of Integrative Bioscience and Biotechnology, Sejong University, Seoul, 05006 Republic of Korea; 6 Present Address: Department of Biology Education, Korea National University of Education, Cheongju, 28173 Republic of Korea

## Abstract

Cranial irradiation is the main therapeutic strategy for treating primary and metastatic brain tumors. However, radiation is well-known to induce several unexpected side effects including emotional disorders. Although radiation-induced depression may cause decreased quality of life after radiotherapy, investigations of its molecular mechanism and therapeutic strategies are still insufficient. In this study, we found that behavioral symptoms of depression on mice models with the decrease of BrdU/NeuN- and Dcx-positive populations and MAP-2 expression in hippocampus were induced by cranial irradiation, and transthyretin (TTR) was highly expressed in hippocampus after irradiation. It was shown that overexpression of TTR resulted in the inhibition of retinol-mediated neuritogenesis. PAK1 phosphorylation and MAP-2 expression were significantly reduced by TTR overexpression following irradiation. Moreover, we observed that treatment of allantoin and neferine, the active components of *Nelumbo nucifera*, interrupted irradiation-induced TTR overexpression, consequently leading to the increase of PAK1 phosphorylation, neurite extension, BrdU/NeuN- and Dcx-positive populations, and MAP-2 expression. Behavioral symptoms of depression following cranial irradiation were also relieved by treatment of allantoin and neferine. These findings demonstrate that TTR plays a critical role in neurogenesis after irradiation, and allantoin and neferine could be potential drug candidates for recovering the effects of radiation on neurogenesis and depression.

## Introduction

Around 75% of patients with solid malignant tumors receive radiation therapy with curative or palliative intent at some point in the course of their disease. However, early and late side effects limit radiation dose and might affect the patient’s health-related quality of life. Although cranial radiation is the main therapeutic strategy for treating primary and metastatic brain tumors, ionizing radiation (IR) is known to have detrimental effects on the nervous system *in vivo* and *in vitro*^1,2^. Late delayed brain injuries induced by neurotoxicity, which are observed six months or later after radiotherapy, include vascular abnormalities, demyelination, white matter necrosis, cognitive impairment, and chronic depression. Unlike early symptoms, the late delayed effects are considered progressive and irreversible^3^. Depression and cognitive impairments tend to occur more often in cancer patients than in the general population^4^, suggesting an important relationship between depression and survival among cancer patients. Furthermore, the mortality rate for depressed cancer patients was twice as high as for other cancer patients over 17 years of follow-up^5^, indicating the importance of managing depression during cancer treatment. It is particularly difficult to establish the causes and mechanisms associated with depression following cancer radiotherapy, since cancer diagnosis is associated with highly negative cognitions with regard to treatments, and could affect psychiatric comorbidity prior to cancer therapy^6^. Therefore, experimental models have been careful in their investigations of depressive symptoms as possible side effects of cancer radiotherapy.

Hippocampal neurogenesis is one possible mechanism for the occurrence of emotional problems in experimental animals, because cranial irradiation decreases the rate of neurogenesis in adult rodent hippocampus^7^. Decreased neurogenesis implicated in the pathogenesis of anxiety and depression has been measured by tail suspension test (TST), forced swimming test (FST), and sucrose preference test for anhedonia^8^. Furthermore, several preclinical studies showed that depression-like behaviors were induced in the late phase after cranial irradiation^7,9^. However, a previous study reported no significant alteration in depression-like behavior using the TST, and only modest effects were detected during initiation of the behavioral task^10^. Thus, the appearance of depression-like behavior after cranial irradiation remains controversial, and more comprehensive studies regarding emotional regulation are needed.

Retinol (vitamin A) plays critical roles in both the embryo and the adult, where it regulates multiple cellular processes and is essential to embryonic development, immune function, vision, and neurogenesis^11–13^. Retinol is secreted from storage into the circulation bound to retinol-binding protein (RBP), a 21-kDa polypeptide that contains one binding site for retinol. In most mammals, retinol-bound RBP (holo-RBP) circulates with transthyretin (TTR), a 56-kDa homotetramer that functions as a carrier for thyroid hormones^14,15^. It is believed that holo-RBP-TTR complex formation prevents loss of the smaller protein from blood by filtration in the glomeruli. Association with the RBP-TTR complex allows the poorly soluble retinol to circulate in blood, but the vitamin dissociates from RBP prior to entering cells. Retinol and its main metabolite, retinoic acid (RA), play key roles in hippocampal neurogenesis, anxiety-like behavior, and hippocampus-dependent memory during adulthood^16^. Moreover, disruption of the retinol signaling pathway has been shown to be involved in age-related memory decline, hippocampal long-term potentiation, and neurogenesis^17,18^. Although several studies have been conducted to investigate the association between retinol-mediated signaling and adult neurogenesis, little is known about the role of regulation of retinol signaling mediated by TTR and RBP in radiation-induced depressive symptom.

To elucidate the molecular mechanism and key regulators involved in radiation-induced emotional disorder, we screened for changes in the transcriptome of brain tissues of mice with depressive symptoms following irradiation. Radiation-induced increases in TTR expression were shown to play important roles in retinol uptake of neuroblastoma cells and subsequent neurogenesis. Furthermore, active ingredients of natural products were shown to have anti-depressive effects. We suggest that our studies will provide therapeutic strategies for IR-induced depression, and help elucidate the possible molecular mechanisms of radiation-induced emotional disorder.

## Results

### Depressive symptoms after cranial irradiation in mouse models

**Figure 1:**
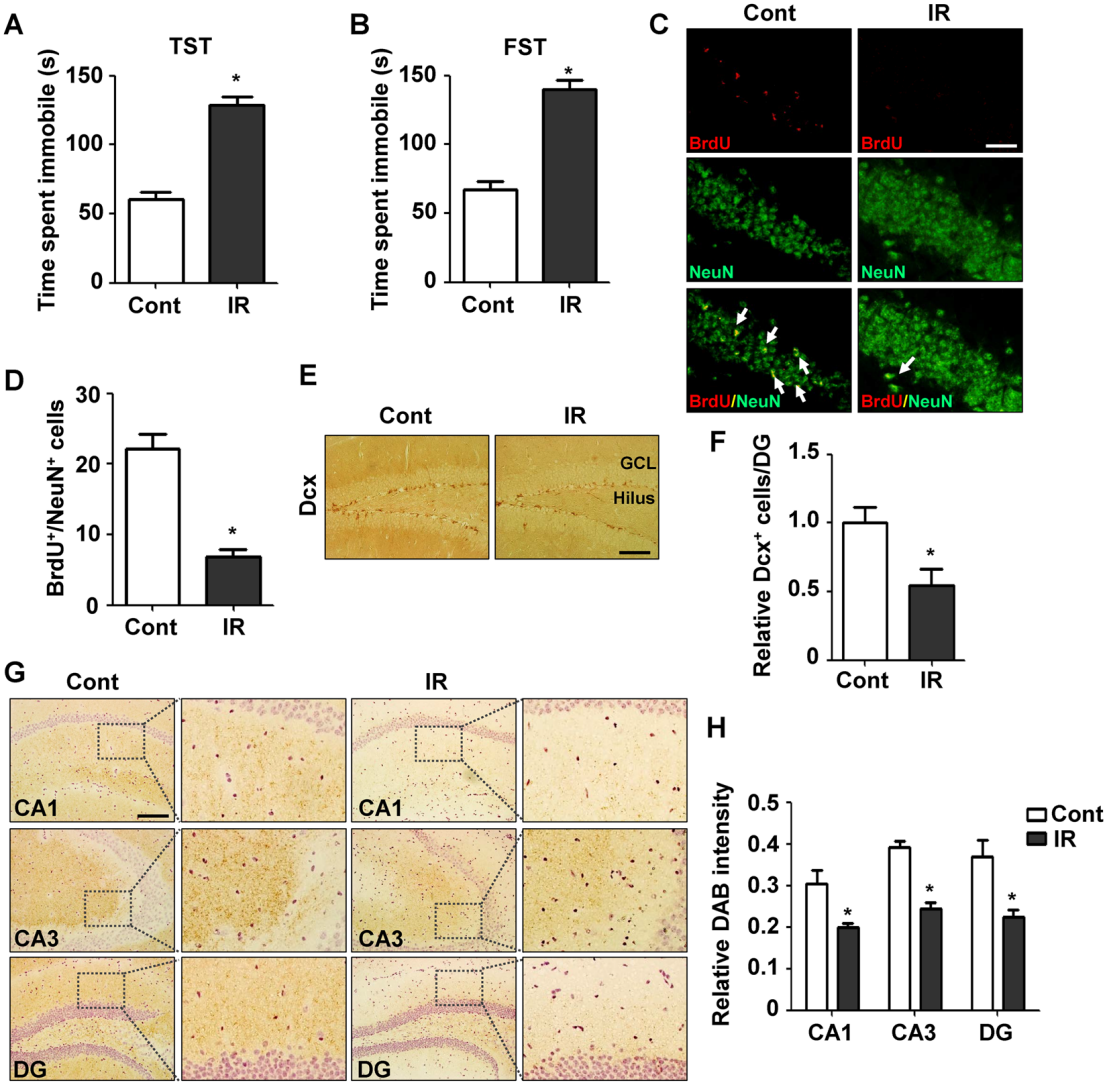
Depressive symptoms after cranial irradiation were investigated by mouse models. (**A**) Depressive behavior after cranial irradiation was assessed by TST. C57BL/6 mice were treated with cranial radiation and duration of immobility was checked after 30 days. (**B**) Depressive symptom after cranial irradiation was analyzed by FST. (**C**) The effects of cranial irradiation on neurogenesis in hippocampal regions were analyzed by immunohistochemistry (IHC). C57BL/6 mice were with cranial radiation and sacrificed after 30 days. BrdU and NeuN were stained in red and green, respectively, and double-positive cells were pointed by arrows in merged images. The magnification was ×200. Scale bar is 25 μm. (**D**) The double-positive cells with BrdU and NeuN in IHC were counted in randomly selected 5 fields of DG and statistically analyzed. *p < 0.05 vs. control mice. (**E**) The effects of cranial irradiation on Dcx expression in hippocampal regions were analyzed by IHC (GCL-Granule Cell Layer). Scale bar is 50 μm. (**F**) The Dcx-stained cells in IHC was counted in the whole area of DG and statistically analyzed. *p < 0.05 vs. control mice. (**G**) The effects of cranial irradiation on MAP-2 expression were analyzed by IHC (CA1-Cornu Ammonis 1; CA3-Cornu Ammonis 3; DG-dentate gyrus). The magnification of images was ×200 (left) and ×400 (right), respectively. Scale bar is 50 μm. (**H**) The MAP-2 staining intensity in IHC was quantified and analyzed by ImageJ/Fiji program. *p < 0.05 vs. control mice.

To determine the effects of cranial radiation on depressive symptoms, we first examined the behavioral outcomes of cranial radiation in mouse models. Depression-like behaviors in adult C57BL/6 mice were assessed using the TST and FST at 30 days after a single cranial exposure to 2 Gy of radiation. Mice exposed to radiation exhibited significantly increased duration of immobility in TST at 30 days after irradiation (128.4 ± 6.66, n = 20, *p*-value < 0.0001), when compared to the non-irradiated control group (60.34 ± 5.48, n = 20) (Fig. 1A). Furthermore, active twisting movements with curling were significantly reduced by irradiation and passive swaying time was increased when the mice were in mobile. In FST, the irradiated mice showed significantly increased duration of immobility at 30 days after irradiation (139.9 ± 6.66, n = 20, *p*-value < 0.0001), relative to the non-treated group (67.47 ± 5.54, n = 20) (Fig. 1B). The climbing activities and swimming of submerged mice were also reduced by cranial irradiation. Taken together, these data indicate that cranial irradiation induces behavioral dysfunction in mouse depression models. Because both the TST and FST are hippocampus-related behavioral paradigms^19^ and the hippocampus is a well-known key regulator of emotion^20^, we next investigated the effects of cranial radiation on hippocampal neurogenesis. When we double-stained BrdU and NeuN, which indicate new born neurons, BrdU^+^/NeuN^+^ population in dentate gyrus (DG) was significantly reduced at 30 days after cranial irradiation (Fig. 1C,D). In addition, doublecortin (Dcx)-positive cells, an immunohistochemical marker of neurogenesis, in DG regions was remarkably decreased by irradiation (Fig. 1E,F). MAP-2 is well known to be abundantly expressed in soma and dendrites of neuronal cells. Since MAP-2 plays a crucial role in microtubule stabilization and neural plasticity, de-regulated expression of MAP-2 is an indicator for neuronal degeneration following brain injury^21,22^. As shown in Fig. 1G,H, the expression levels of MAP-2 were significantly decreased in cornu ammonis 1 (CA1), CA3, and DG of the hippocampal region, which indicated dendritic damage in hippocampal region by irradiation. These observations show that cranial radiation represses adult neurogenesis in the hippocampal regions, which could be a possible cause of depression-like behavior after cranial irradiation.

### TTR was selected as a candidate to induce depressive symptoms by irradiation and increased by irrad-iation *in vitro* and *in vivo*

**Figure 2:**
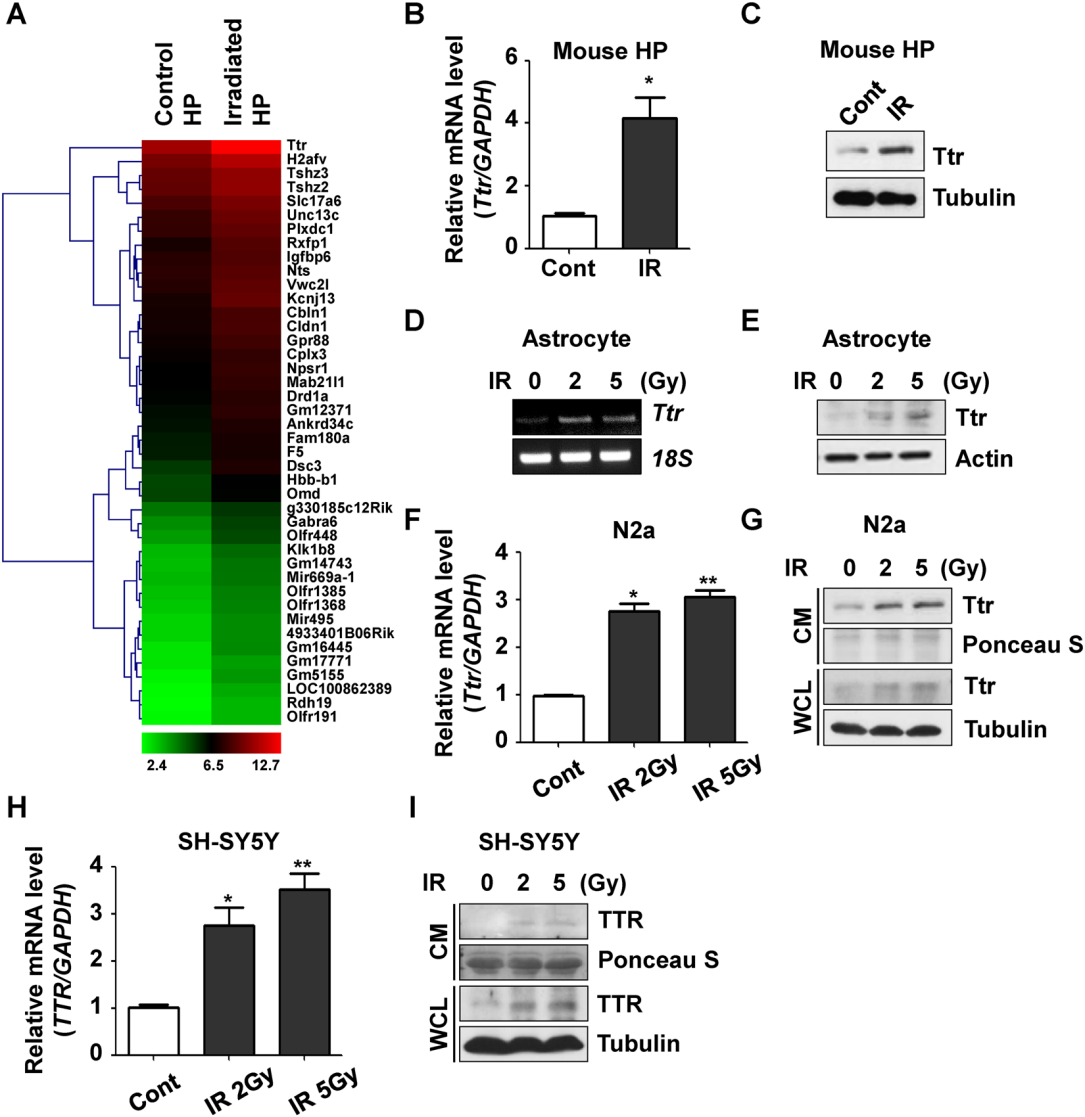
TTR was selected as a candidate to induce depressive symptoms by irradiation and increased by irradiation *in vitro* and *in vivo*. (**A**) Heat map representing gene expression changes (at least 2-fold) in mouse hippocampus following cranial irradiation. Microarray experiments were performed using cDNA samples obtained from hippocampus tissues treated or non-treated with 2 Gy of radiation. The microarray results were deposited in the Gene Expression Omnibus database (GSE94440). (**B**) mRNA levels of Ttr after irradiation were analyzed by real-time qRT-PCR in mouse hippocampus tissues. (**C**) The effects of irradiation on Ttr protein expression in mouse hippocampal regions were confirmed by western blot analysis. (**D**) mRNA levels of *Ttr* after irradiation were analyzed by RT-PCR in primary cultured mouse astrocytes and normalized using the level of *18S* as an internal control. Astrocytes were treated with 0, 2, and 5 Gy of radiation and incubated for 24 h. (**E**) The effects of irradiation on Ttr protein expression in primary cultured mouse astrocytes were confirmed by western blot analysis. Actin was used as an internal control. (**F**) Expression levels of *Ttr* in N2a cells were analyzed by real-time qRT-PCR. N2a cells were treated with 0, 2, and 5 Gy of radiation and incubated for 24 h, after which total RNA was used to investigate the effects of irradiation on *Ttr* levels. ^*^p < 0.05 vs. non-irradiated cells. (**G**) The effects of irradiation on Ttr protein expression in N2a cells were confirmed by western blot analysis. Cells were irradiated with 0, 2, and 5 Gy of radiation, then incubated for 24 h. CM and whole cell lysates were used to investigate the external and the internal levels of Ttr, respectively. Ponceus S staining and the levels of tubulin were checked for equal quantification of each sample. (**H**) Expression levels of *TTR* in SH-SY5Y cells were analyzed by real-time qRT-PCR. ^*^p < 0.05 vs. non-irradiated cells. (**I**) The effects of irradiation on TTR protein expression in SH-SY5Y cells was confirmed by western blot analysis.

To elucidate targets that might be regulated by cranial irradiation in the hippocampus, we isolated total RNA from mice hippocampuses treated or not-treated with 2 Gy of cranial radiation. RNA samples were then subjected to microarray analysis. Among the 52 coding genes up- or down-regulated by more than 2-fold by cranial irradiation deposited in the Gene Expression Omnibus database (GEO Series accession number GSE94440), TTR showed the greatest increase of about 4-fold and was therefore selected for further studies (Fig. 2A). To verify the microarray data, we investigated mRNA and protein levels of Ttr in mouse hippocampus after cranial irradiation. As shown in Fig. 2B,C, the expression of Ttr in hippocampus was significantly increased by irradiation in mRNA and protein levels. In addition, since TTR is associated with regulation of retinol uptake and astrocytes are major components with high composition ratio to consist of blood-brain barrier contributing to the interaction between neurons and blood^23^, we checked the levels of Ttr in primary cultured astrocytes from the mouse hippocampus. After treatment with 2 and 5 Gy of radiation, mRNA and protein levels of Ttr were increased, respectively (Fig. 2D,E). N2a cells and SH-SY5Y cells were used to confirm the *ex vivo* data and to study neuronal differentiation and neurogenesis *in vitro*. As shown in Fig. 2F,G, the mRNA and protein levels of Ttr in N2a cells were elevated by irradiation, respectively. In parallel, the expression levels of TTR in SH-SY5Y cells were significantly increased by irradiation (Fig. 2H,I). As previously described, TTR is a circulating molecule that is secreted after synthesis, so we checked the secreted levels of TTR in conditioned medium (CM) after irradiation. Interestingly, the secreted levels of TTR of N2a and SH-SY5Y cells were increased by irradiation (Fig. 2G,I). To identify whether TTR expression could also be affected by low-dose radiation (LDR), we investigated alterations in TTR expression in the SH-SY5Y and N2a cell lines. The protein expression of TTR was not increased by dose-dependent exposure to LDR (Fig. S1A,B). Moreover, the mRNA level of TTR was not affected by irradiation with 10, 50, 100 mGy of radiation in either cell lines (Fig. S1C,D) indicating that cranial exposure to LDR could not increase TTR expression. These results indicate that cranial irradiation increases the expression and subsequent secretion of TTR *in vitro* and *in vivo*.

### TTR decreased retinol-mediated neurite outgrowth and uptake of retinol

**Figure 3:**
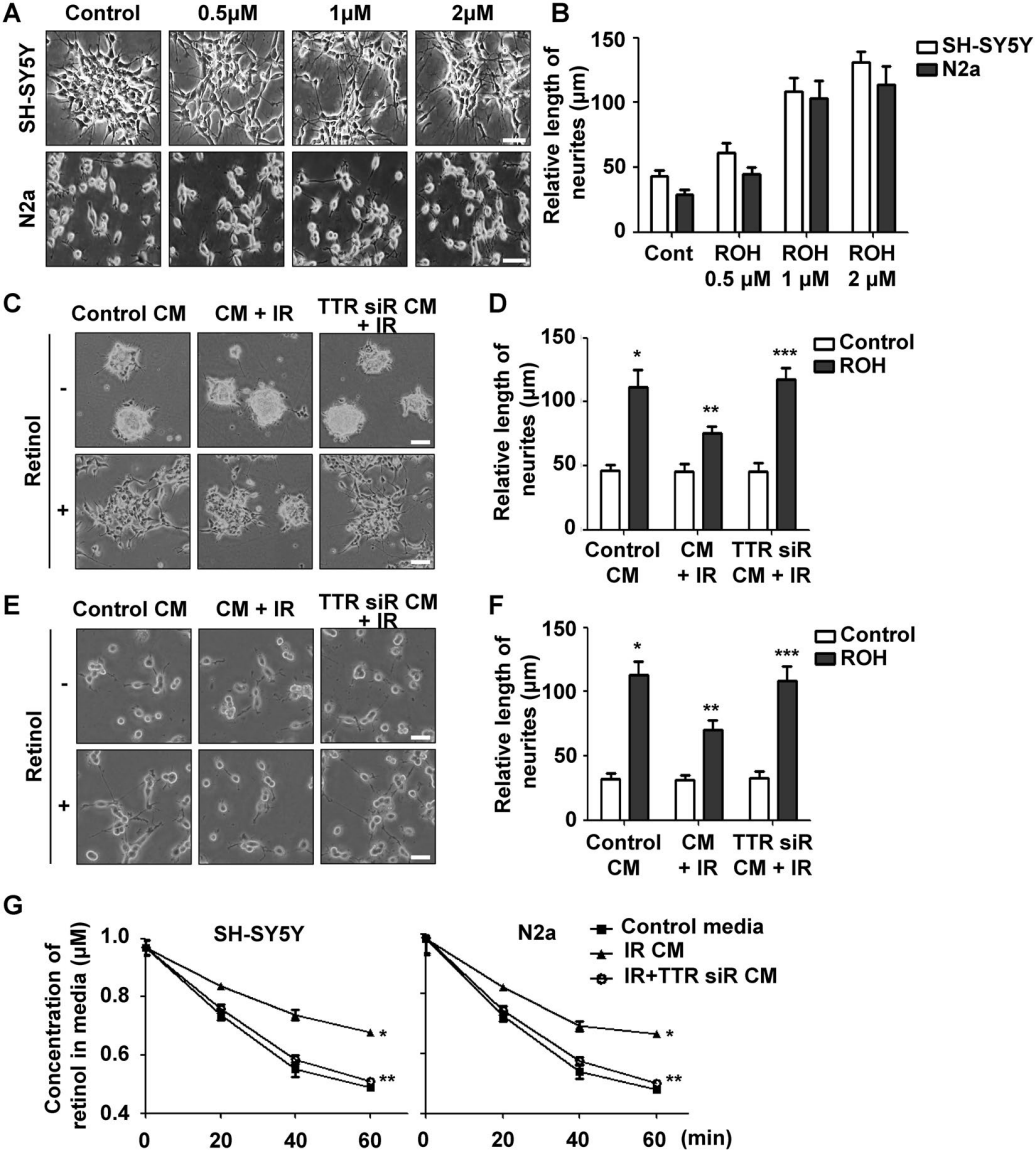
TTR decreased retinol-mediated neurite outgrowth and uptake of retinol. (**A**) The effects of retinol on neurite outgrowth were investigated by morphological changes in SH-SY5Y and N2a cell lines following retinol treatment. Both cells were treated with 0, 0.5, 1, and 2 μM of retinol, then incubated for 24 h. Scale bar is 100 μm. (**B**) The retinol-mediated neurite outgrowth was quantified by measuring the length of neurites. (**C**) The effects of TTR on retinol-mediated neuritogenesis in SH-SY5Y cells were analyzed by microscopic examination. Cells were treated with CM from non-treated, irradiated, and TTR siRNA + IR treated cells. At 24 h following treatment with 1 μM of retinol, morphological alteration of SH-SY5Y cells was checked. (**D**) The effects of TTR on retinol-mediated neurite outgrowth in SH-SY5Y cells were quantified by measuring the length of neurites and statistically analyzed. *p < 0.05 vs. cells with control CM; **p < 0.05 vs. cells with CM + IR; ***p < 0.05 vs. cells with TTR siRNA CM + IR. (**E**) The effects of Ttr on retinol-mediated neuritogenesis in N2a cells were analyzed by microscopic examination. (**F**) The effects of Ttr on retinol-mediated neurite outgrowth in N2a cells were quantified by measuring the length of neurites and statistically analyzed. *p < 0.05 vs. cells with control CM; **p < 0.05 vs. cells with CM + IR; ***p < 0.05 vs. cells with TTR siRNA CM + IR. (**G**) The inhibitory effects of extracellular TTR on retinol uptake in SH-SY5Y and N2a cell lines were examined by ELISA. Both cells were incubated with retinol and CM extracted from non-treated, irradiated, and TTR siRNA + IR treated cells. The concentration of retinol in media was time-dependently analyzed using a retinol detecting ELISA kit. *p < 0.001 vs. cells with control CM; **p < 0.001 vs. cells with CM + IR.

TTR is a key regulator of retinol transportation and retinol play pivotal roles in hippocampal neurogenesis^14,15^. Therefore, it has been suggested that TTR carries the RBP-retinol complex to brain regions, which mediate retinol-induced neurite outgrowth and neurogenesis. However, the uptake of retinol is mediated by binding of holo-RBP to stimulated by retinoic acid 6 (STRA6) at target cells. Berry D.C. and his colleagues recently reported that TTR could block retinol uptake by inhibiting the contact between holo-RBP and STRA6^24^. To investigate the effects of retinol-mediated neurogenesis, we first tested retinol-induced neurite outgrowth. As shown in Fig. 3A,B, retinol induces axonal outgrowth and extension dose dependently in N2a and SH-SY5Y cells. To determine whether the increase in TTR by irradiation inhibits retinol-mediated neurite outgrowth, we extracted CM from N2a and SH-SY5Y cells treated or non-treated with IR and TTR-specific siRNA. When treated with CM from the irradiated cells, neurite extension of both cells mediated by retinol treatment was significantly reduced compared to cells treated with CM from the non-treated cells (Fig. 3C–F). Furthermore, reduced neuritogenesis by CM from the irradiated cells was recovered similarly to the control group by treatment of CM from irradiated cells transfected with TTR siRNA. When it comes to LDR exposure, retinol-mediated neurite outgrowth was not blocked by LDR (Fig. S1E–H). To clarify whether increased TTR inhibits retinol uptake, we measured the levels of retinol dissolved in the culture media after 1 h following retinol treatment. The concentration of retinol in the media was reduced more slowly when cells were treated with CM from the irradiated cells than when they were treated with control media (Fig. 3G). Conversely, the concentration of retinol was reduced again when cells were treated with CM obtained from irradiated cells transfected with TTR siRNA. Taken together, these findings suggest that cranial irradiation might induce depressive symptoms through inhibition of retinol-mediated neuritogenesis via the increase in TTR in response to radiation.

### Neuritogenesis after retinol uptake was mediated by the RAR-Rac1-PAK1 signaling axis

**Figure 4:**
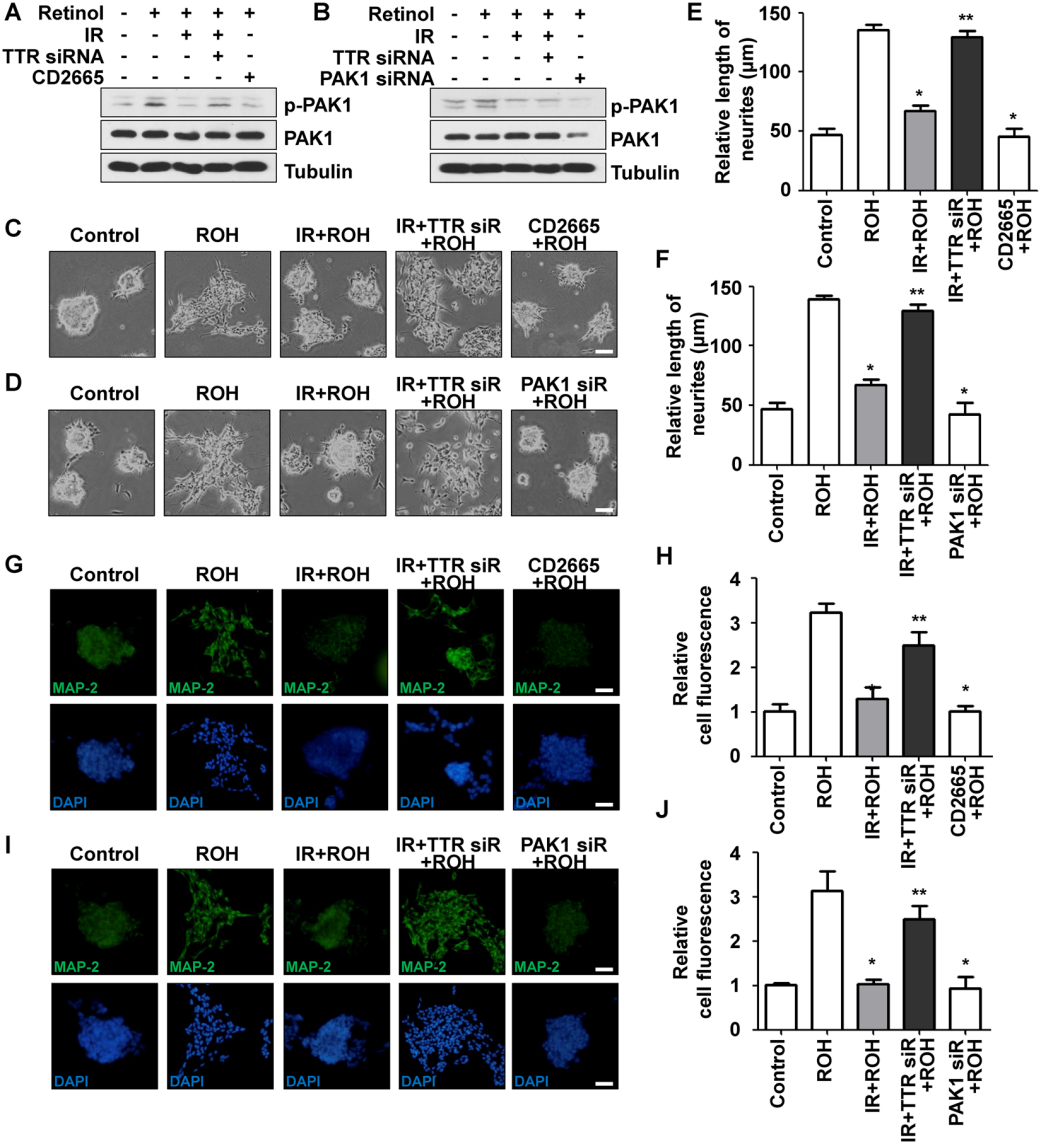
Neuritogenesis after retinol uptake was mediated by the RAR-Rac1-PAK1 signaling axis. (**A**) The inhibitory effects of TTR on retinol-mediated PAK1 phosphorylation in SH-SY5Y cells were investigated by Western blot analysis. SH-SY5Y cells were transfected with control siRNA or TTR siRNA for 48 h, then irradiated or treated with CD2665. At 24 h after retinol treatment, cells were trypsinized and lysates were used for analysis. Tubulin was used as an internal control. (**B**) The effects of TTR on retinol-mediated PAK1 phosphorylation in SH-SY5Y cells were confirmed using PAK1-specific siRNA. (**C**) The effects of TTR-mediated PAK1 inhibition on neurite outgrowth were investigated by microscopic observation. Scale bar is 100 μm. (**D**) The effects of TTR-mediated PAK1 inhibition on neurite outgrowth were confirmed by knockdown of PAK1 using specific siRNA. (**E**) The effects of TTR-mediated PAK1 inhibition on neurite outgrowth were quantified by evaluating the length of neurites and statistically analyzed. *p < 0.05 vs. retinol-treated cells. **p < 0.05 vs. irradiated and retinol-treated cells. (**F**) The effects of TTR-mediated PAK1 inhibition on neurite outgrowth were quantified by measuring the length of neurites and statistically analyzed. *p < 0.05 vs. retinol-treated cells. **p < 0.05 vs. irradiated and retinol-treated cells. (**G**) The effects of TTR-mediated PAK1 inhibition on MAP-2 expression were investigated by immunocytochemistry. (**H**) The effects of TTR-mediated PAK1 inhibition on MAP-2 expression were confirmed by using PAK1-specific siRNA. (**I**) The effects of TTR-mediated PAK1 inhibition on MAP-2 expression were quantified and analyzed by ImageJ/Fiji program. *p < 0.05 vs. retinol-treated cells. **p < 0.05 vs. irradiated and retinol-treated cells. (**J**) The effects of TTR-mediated PAK1 inhibition on MAP-2 expression were quantified and analyzed. *p < 0.05 vs. retinol-treated cells. **p < 0.05 vs. irradiated and retinol-treated cells.

Retinol is delivered by TTR and RBP in normal condition via translocation into cells^15^. In cytosol, retinol is converted into RA, which induces neurogenesis by binding to RA receptor (RAR) following conversion. The RA-RAR complex has been reported to activate Rac1^25^, but the involvement of PAK1, its key downstream effector, in this signaling axis has not been thoroughly investigated. Therefore, we investigated whether phosphorylation of PAK1 was increased by retinol treatment and decreased by irradiation, which increases TTR expression. The PAK1 phosphorylation increased by retinol treatment was significantly decreased by irradiation in the SH-SY5Y and N2a cell lines, respectively (Figs 4A,B, S2A,B). When both cell lines were transfected with TTR siRNA, the decreased level of phospho-PAK1 after treatment with retinol and radiation was recovered, although phosphorylated PAK1 was downregulated by treatment of CD2665, a Rac1 antagonist, and PAK1-specific siRNA used as a positive inhibitor control of PAK1 signaling. To determine if PAK1 signaling plays important roles in neuritogenesis of both cell lines, we investigated the morphological alterations and expression levels of MAP-2 through microscopic observations. Neurite outgrowth promoted by retinol treatment was reduced by radiation pre-treatment and recovered by transfection of TTR siRNA in SH-SY5Y and N2a cells (Figs 4C–F and S2C–F). However, neurite extension was significantly disrupted by CD2665 treatment and PAK1 siRNA transfection. Moreover, MAP-2 expression elevated by retinol treatment was significantly reduced by irradiation, then increased again following knockdown of TTR, although its expression was decreased by CD2665 treatment and PAK1 knockdown (Figs 4G–J and S2G–J). In addition to MAP-2 expression, morphology with extended neurites after retinol treatment was reduced by irradiation and recovered by TTR siRNA treatment. These data indicate that the increase in TTR after irradiation inhibits retinol-mediated activation of PAK1 signaling, which disrupts neurite outgrowth and neurogenesis.

### Allantoin and neferine rescued IR-induced decrease of neurogenesis

**Figure 5:**
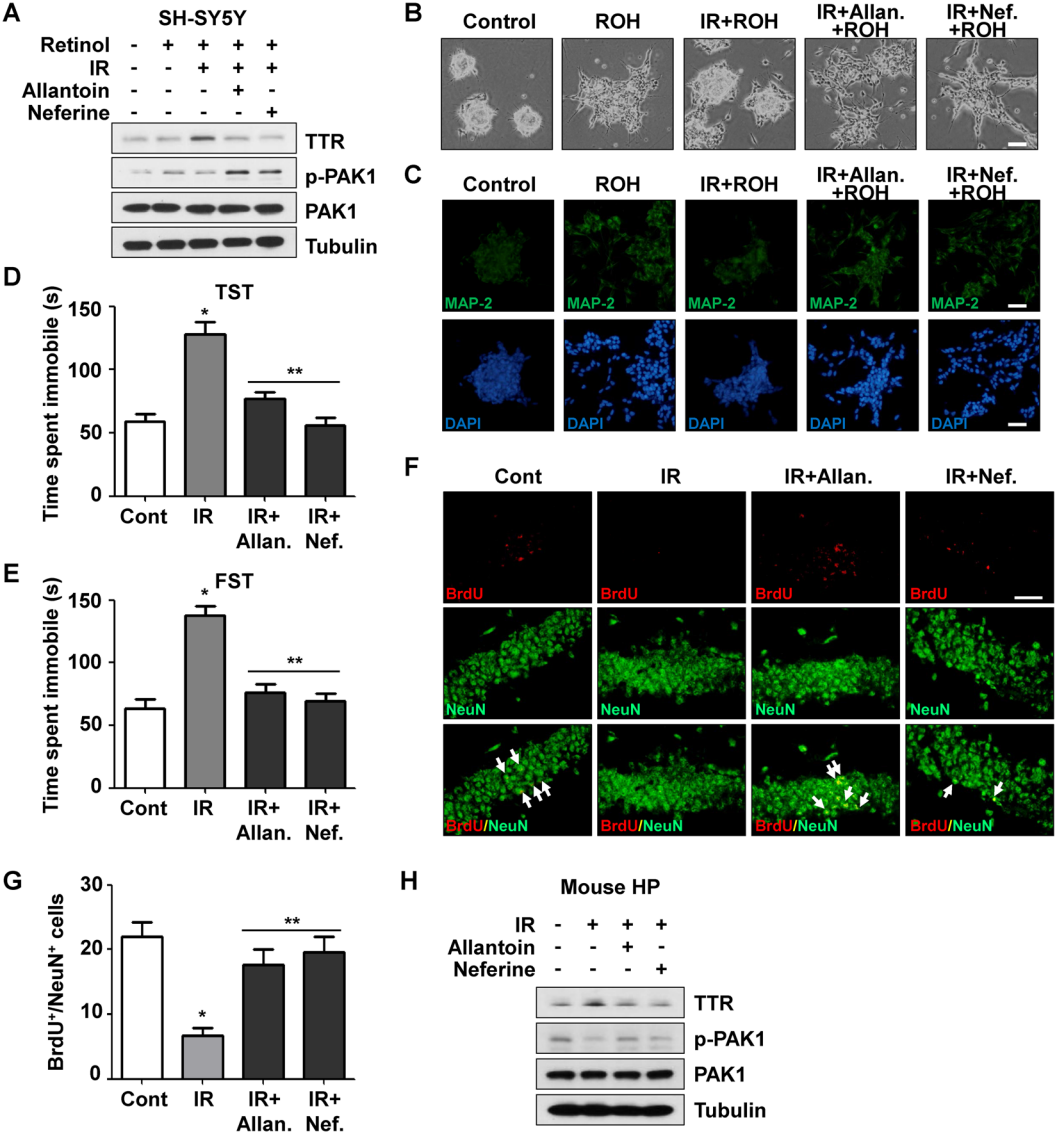
Allantoin and neferine rescued IR-induced decrease of neurogenesis and depression-like symptoms. (**A**) The effects of both allantoin and neferine on TTR expression and PAK1 phosphorylation in SH-SY5Y cells were analyzed by western blot assay. SH-SY5Y cells were treated with 10 μM of allantoin or 2 μM of neferine with irradiation and incubated for 24 h. After 24 h following treatment of retinol, cells were trypsinized and lysates were used for analysis. Tubulin was utilized for equal quantification. (**B**) The effects of TTR inhibition by allantoin and neferine on IR-mediated repression of neurite outgrowth were analyzed by microscopic observation. Scale bar is 100 μm. (**C**) The effects of TTR inhibition by allantoin and neferine on IR-mediated repression MAP-2 expression were investigated by immunocytochemistry. (**D**) Depressive behavior after cranial irradiation was assessed by TST. C57BL/6 mice were treated with cranial radiation and injected intraperitoneally with 30 mg/kg of allantoin and 15 mg/kg of neferine per 3 days. Duration of immobility was checked after 30 days. *p < 0.05 vs. control mice. **p < 0.05 vs. irradiated mice. (**E**) Depressive symptom after cranial irradiation was analyzed by FST. *p < 0.05 vs. control mice. **p < 0.05 vs. irradiated mice. (**F**) The effects of allantoin and neferine on neuritogenesis after cranial irradiation in hippocampal regions were analyzed by immunohistochemistry. BrdU and NeuN were stained in red and green, respectively, and double-positive cells were pointed by arrows in merged images. Scale bar is 25 μm. (**G**) The double-positive cells with BrdU and NeuN in IHC were counted in randomly selected 5 fields of DG and statistically analyzed. *p < 0.05 vs. control mice. **p < 0.05 vs. irradiated mice. (**H**) The effects of allantoin and neferine on TTR expression and PAK1 phosphorylation in mouse hippocampus were analyzed by Western blot analysis.

*Nelumbo nucifera* is an aquatic plant commonly known as the lotus. The rhizome of *Nelumbo nucifera* is known for its anti-depressive effects, and allantoin and neferine were known for representative bioreactives in the rhizome and reported to relieve depressive symptoms^26,27^. Allantoin is a product of oxidation of uric acid and has antidiabetic effects^28^. Neferine has various effects including anti-fibrotic effects, inhibition of cell proliferation, and autophagy induction^29–31^. However, neither of these compounds has been investigated for neurogenic effects; therefore, they were investigated to determine if they could rescue the reduction in neurogenesis caused by irradiation. We first examined the effects of allantoin and neferine on the expression of TTR and activation of PAK1 signaling. As previously described, the level of TTR was increased and phosphorylation of PAK1 was decreased by irradiation of SH-SY5Y and N2a cells (Figs 5A and S3A). However, TTR expression decreased and PAK1 phosphorylation increased significantly in response to treatment with allantoin and neferine. We next confirmed the effects of allantoin and neferine on neuronal maturation and MAP-2 expression following irradiation. As shown in Figs 5B, S3B–E, decreased neurite expansion following irradiation was significantly recovered by treatment with allantoin and neferine. SH-SY5Y and N2a cells showed a greater distribution pattern with elongated neurites after treatment of both components. Furthermore, the expression of neuronal marker, MAP-2, increased significantly in response to allantoin and neferine treatment in both cell lines (Figs 5C, S3C–G). Taken together, these findings demonstrated that allantoin and neferine reinforced retinol-mediated neuronal maturation through the inhibition of TTR expression and promotion of PAK1 phosphorylation.

### Allantoin and neferine recovered IR-induced depressive symptoms in mouse model

To investigate whether allantoin and neferine could relieve IR-induced depressive symptoms *in vivo*, we injected allantoin, neferine, or vehicle (DMSO) intraperitoneally after cranial irradiation, then checked the time for which the mice remained immobile by the TST and FST. Mice treated with cranial radiation showed significantly increased duration of immobility in the TST at 30 days after irradiation (128.3 ± 9.65, n = 20, *p*-value < 0.0001) relative to non-irradiated control group (59.00 ± 5.78, n = 20) (Fig. 5D). Interestingly, treatment with allantoin and neferine reduced the immobility to levels similar to the control group (76.89 ± 5.42 and 55.66 ± 5.42, respectively, n = 20, *p*-value < 0.0001). The reduction in active twisting movements in response to cranial irradiation was recovered and increased passive swaying time was reduced in response to allantoin and neferine injection. Moreover, in the FST, the immobility extended by cranial irradiation (138.1 ± 7.17, n = 20, *p*-value < 0.0001) was significantly decreased by periodic treatment of both components (76.21 ± 6.49 and 69.71 ± 5.86, respectively, n = 20, *p*-value < 0.0001) (Fig. 5E). The reduction in climbing activities and swimming of submerged mice increased following allantoin and neferine treatment. These data indicate that allantoin and neferine alleviate the depressive symptoms that occur following cranial irradiation *in vivo*. To understand the effects of allantoin and neferine on neurogenesis and neurodegeneration in the hippocampus, we investigated the population of BrdU^+^/NeuN^+^ and Dcx^+^ cells, and the expression levels of MAP-2 in hippocampal regions. As shown in Fig. 5F,G, the decrease of BrdU^+^/NeuN^+^ population after cranial irradiation was remarkably increased by treatment of allantoin or neferine. The Dcx-positive population was also reduced by irradiation and recovered by treatment of allantoin or neferine (Fig. S4A,B). The decreased levels of MAP-2 in CA1, CA2 and DG in response to cranial irradiation were significantly increased by treatment with allantoin or neferine (Fig. S4C,D). To confirm whether the overexpression of TTR and subsequent PAK1 signaling inactivation was also regulated by both compounds *in vivo*, we investigated TTR protein expression and PAK1 signaling activation in mouse hippocampus. As shown in Fig. 5H, the expression of TTR in hippocampus was increased after irradiation and significantly decreased by treatment of allantoin and neferine. Conversely, the phosphorylation of PAK1 was decreased by irradiation and recovered by treatment of both compounds (Fig. 5H). Overall, these findings indicate that allantoin and neferine could mitigate the depression-like symptoms induced by irradiation by enhancing hippocampal neurogenesis and recovering neurodegeneration through the inhibition of TTR expression.

## Discussion

Depression is the second leading cause of disability worldwide and adversely affects the course and outcome of chronic conditions such as cancer, diabetes and obesity^32,33^. It is estimated that approximately 10% to 30% of cancer patients have a major depressive disorder^34^. Although depression is a complex neuropsychiatric disease with a poorly defined etiology, the possibility that altered hippocampal neurogenesis in adult is fundamentally involved in the pathophysiology of depressive symptoms has been suggested by several previous studies^35–37^. These studies have shown that adult-generated hippocampal neurons are needed for proper mood control and for antidepressant activity. However, some studies have reported conflicting results regarding the validity of this neurogenic hypothesis of depression. In laboratory animals, ablation of neurogenesis does not always cause depressive-like symptoms^38^, stress does not always decrease neurogenesis^39^, and some effects of antidepressants are independent to neurogenesis^40^. Our results suggested a possible explanation describing specific mechanisms of neurogenic disruption by irradiation that might induce depressive symptoms. We observed that radiation-induced TTR overexpression significantly reduced cellular uptake of retinol and subsequent neurite outgrowth *in vitro* (Fig. 3). Moreover, inhibition of TTR overexpression recovered neurite expansion and expression of mature neuronal markers, which eventually reduced immobility in behavioral mouse models and increased hippocampal neurogenesis in the mouse brain (Fig. 5). Collectively, these findings suggest that depression that appears as a side effect of cranial radiotherapy might be caused by the inhibition of retinol-mediated adult neurogenesis in hippocampal regions.

TTR is well-known to form a complex with holo-RBP and retinol thus reaches target tissues bound in holo-RBP-TTR complex. However, as shown in Fig. 2 and 3, secretion of TTR increased by irradiation led to reduction of retinol uptake and neurite outgrowth *in vitro*. This discrepancy might be explained by our results, as well as those of several supporting studies describing the mechanism of retinol uptake into the target cells. It has been suggested that uptake of retinol from circulating holo-RBP is mediated by its receptor, STRA6^41–43^. STRA6 binds to holo-RBP and subsequently transports retinol into cells^43^. In adults, STRA6 has been reported to be highly expressed in cells that compose blood-organ barriers in various organs, including the brain^44^. Therefore, uptake of retinol into the brain regions might be mediated by binding of holo-RBP to STRA6. Human holo-RBP (PDB ID: 1RBP) consists of eight antiparallel β-strands, an N-terminal coil, and a short α-helix close to the C-terminus. The eight-stranded β-barrel forms a cavity where the retinol is encapsulated inside. According to the structural evidences, three ligand-entrance loops of β-barrel including the residues 31–38, 63–67, and 93–99 in holo-RBP are suggested to be principally involved in the formation of complex with TTR (PDB ID: 1QAB)^45,46^. In addition, antiparallel β-barrel of holo-RBP can also interact with extracellular RBP-binding domain of STRA6 located between transmembrane domain VI and VII, leading to transportation of retinol into the cells^47^. These investigations indicate that binding of TTR to holo-RBP can be a structural hindrance to the interaction of holo-RBP with STRA6 in order to transport retinol into the cells. Furthermore, when we checked the raw data of microarray including total gene expression changes, the expression of STRA6 in hippocampus was not changed meaningfully (fold change of STRA6 after irradiation, 1.110; data not shown) whereas TTR was increased about 4-fold after cranial irradiation. Taken together, we carefully concluded that an abnormal increase of TTR and consequently extracellular sequestration of holo-RBP by complex formation with TTR might inhibit retinol uptake by blocking the interaction of holo-RBP with STRA6 in hippocampal region, leading to disruption of retinol-mediated adult hippocampal neurogenesis.

During or after radiation therapy, cancer patients suffering from depressive symptoms have been additionally prescribed with selective serotonin reuptake inhibitors (SSRIs), the most common antidepressants. Although these inhibitors alleviate the symptoms effectively, various side effects have been reported, including sexual dysfunction, several psychiatric problems (suicidal thoughts and manic switching), and even persistent pulmonary hypertension in offspring^48–50^. In addition, it is not that SSRIs could directly recover defective neurogenesis after cancer radiotherapy in order to cure the depression. Therefore, further studies are needed to identify additional molecules to effectively inhibit the pathogenesis of depression. Several previous studies have shown that some natural products have therapeutic effects on Alzheimer’s disease (AD), the representative neurodegenerative disorder, by repressing the key regulators including acetylcholine protease (AChE) and tau^51–53^. Galantamine (Razadyne^®^), an alkaloid isolated from *Galanthus woronowii*, was reported to inhibit AChE activity and maintain acetylcholine levels in AD patients^52^. Few side effects of galantamine were reported and the efficacy and tolerability of galantamine were comparable with other synthetic AChE inhibitors (Rivastigmine and Donepezil), which led to FDA approval in 2001. Minocycline, a synthetic derivative of tetracycline isolated from *Streptomyces* spp., was recently reported to reduce the levels of hyperphosphorylated tau and be tested in a Phase II clinical trial in AD^53^. In the same context with these studies, our present study suggested allantoin and neferine, which are known to be safe and non-toxic^54^, as anti-TTR natural products to relieve depressive symptoms after cranial radiotherapy. Furthermore, the blood-brain barrier (BBB), a highly selective semipermeable protective shield separating the circulating system from the brain extracellular fluid, can become obstacles for development of medicines in neurogenic disorders, including depression. In this reason, any newly discovered compounds to cure the neurogenic diseases must have favorable BBB permeability. Allantoin was reported to cross the intact BBB in rat models and its subcutaneous treatment elevated whole brain allantoin levels (Patent: US 2012/0128654 A1). Neferine was also observed to have BBB permeability and its intravenous injection enhanced the concentration of neferine in mice brain (GTID: 2144360125965306). Collectively, we propose the possibility of allantoin and neferine as potential candidates for treatment of depression via restoration of hippocampal neurodegeneration induced by cranial irradiation.

**Figure 6:**
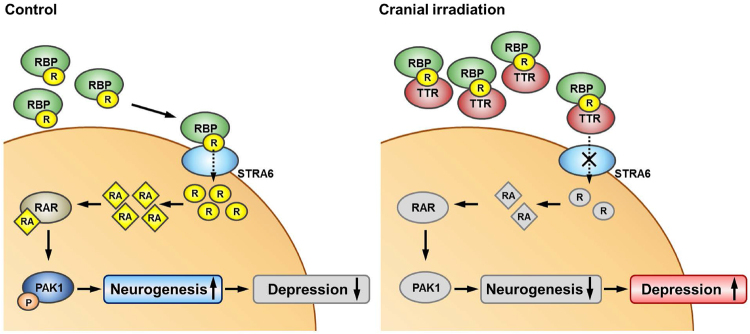
Schematic diagram illustrated that increase of TTR after cranial irradiation inhibits the binding of RBP-STRA6 which leads to intracellular uptake of retinol. Inhibition of retinol uptake decreases RA-mediated activation of RAR-Rac1-PAK1 signaling axis promoting neurogenesis, which consequently induces depression-like symptoms.

Depression is a psychiatric disorder with complex risk factors. Moreover, because of comorbidities and combined treatment regimens with other modalities, such as chemotherapy, it is hard to assess the direct effects of radiation on depression. The results of the present study suggest that TTR increased by irradiation could inhibit the uptake of retinol and its downstream signaling pathway, RAR-Rac1-PAK1 axis, decreasing neurogenesis in the adult hippocampus (Fig. 6). Although the rhizome of *Nelumbo nucifera* has been reported to have antidepressant effects^55^, the investigation of its active constituents is found to be unsatisfactory. We firstly propose that combined treatment of allantoin and neferine with cranial irradiation might be a possible strategy to regulate the occurrence of depressive symptoms by down-regulating the expression of TTR increased by irradiation and activating the PAK1 signaling, which might promote hippocampal neurogenesis.

## Materials and Methods

### Reagents

Retinol, allantoin, and neferine were purchased from Sigma (St. Louis, MO). Cell culture media and fetal bovine serum were acquired from Gibco (Grand Island, NY). Antibodies against Ttr (sc-13098), MAP-2 (sc-20172), α-Tubulin (sc-23948) and β-actin (sc47778) were acquired from Santa Cruz Biotechnology (Santa Cruz, CA). Antibodies specific for BrdU (5292), Dcx (4604), PAK1 (2602) and p-PAK1 (Ser144; 2606) and PAK1 siRNA were acquired from Cell Signaling Technology (Beverly, MA). Antibody for NeuN (ab104224) was purchased from Abcam (Cambridge, UK). RARβ/γ selective antagonist CD2665 was acquired from Sigma (St. Louis, MO).

### Animal protocol

Male C57BL/6 mice (6 weeks of age; Central Lab Animals Inc., Seoul, Republic of Korea) were used for the *in vivo* experiments. The animal protocols were approved by the Institutional Animal Care and Use Committee of Pusan National University (Busan, Republic of Korea), and performed in accordance with the provisions of the NIH Guide for the Care and Use of Laboratory Animals. Mice were housed in groups of up to five in sterile cages. Animals were maintained in animal care facilities in a temperature-regulated room (23 ± 1 °C) under a 12 h light/dark cycle, allocated and randomized for the experiments by the technician of the facility, and quarantined for 1 wk prior to the study. The animals were fed water and a standard mouse chow diet ad libitum.

### Irradiation and tissue sampling

The mice were anesthetized with tiletamine/zolazepam (Zoletil 50^®^; Virak Korea; Seoul, Korea) and immobilized, after which single radiation fractions of 2 Gy was delivered through the whole brain. The brain received whole-brain irradiation using six MV high energy photon rays (ELEKTA; Stockholm, Sweden) at a dose rate of 3.8 Gy/min. A radiation dosimeter (Semiflex Ionization Chamber 31013, PTW Co., Freiburg, Germany) was used to ensure that the radiation doses ranged from 99 to 100% at a point 3 cm below the surface of the simulated mouse head. We aligned the center of the head to the beam line center using the mouse holder. The distance of the skin from radiation source was 1 m. Sham-irradiated mice were anesthetized with tiletamine/zolazepam (Zoletil 50^®^) and immobilized for the same period of time without irradiation. Five mice per group were subjected to TST and FST, followed by brain collection for immunohistochemistry.

### Tail suspension test (TST)

The TST was conducted as previously described^56^. Briefly, mice were suspended from a plastic rod mounted 50 cm above a surface by fastening the tail to the rod with adhesive tape. A 6 min test session was videotaped and immobility scored by 5 trained observers. The average scores measured by 5 observers were used for immobile time of each group.

### Forced swimming test (FST)

The FST was conducted as previously described^19^. In brief, mice were placed in a glass cylinder (height: 30 cm, diameter: 16 cm) containing water at 24 °C and a depth of 14 cm, so that they could neither escape nor touch the bottom. Mice were forced to swim for 6 min. The animals were habituated for the first 1 min and behavior was monitored over the next 5 min. A 6 min test session was videotaped and immobility scored by 5 trained observers. The average scores measured by 5 observers were used for immobile time of each group.

### Immunofluorescence

To observe the population double-stained with BrdU and NeuN in mouse hippocampus tissues and MAP-2 expression in neuronal cell lines, immunofluorescence was used. For BrdU/NeuN immunostaining, brain sections were treated with 50% formamide, 280 mmol/L NaCl and 30 mmol/L sodium citrate at 65 °C for 2 h, incubated in 2 mol/L HCl at 37 °C for 30 min, rinsed in 0.1 mol/L boric acid (pH 8.5) at room temperature for 10 min. After incubating in 3% H_2_O_2_ for 30 min, sections were blocked in PBS containing 2% goat serum, 0.3% Triton X-100, and 0.1% bovine serum albumin for 1 h, followed by incubation with mouse monoclonal anti-BrdU and anti-NeuN antibody at 4 °C overnight. Cells were cultured on the slide glass (Muto. Pure Chemicals Co. Ltd, Tokyo, Japan) and treated with irradiation, CD2665, PAK1 siRNA, allantoin or neferine according to the experiments. The cells were fixed in cold acetone for 10 min at −20 °C and washed with cold PBS twice. The cells were blocked with 1% BSA in PBS was performed for 1 h at room temperature and incubated with primary antibodies at 4 °C for overnight. Next, the cells were washed three times and probed with secondary antibodies conjugated with DyLight 488 or DyLight 594 (Thermo Scientific, Hudson, NH), following staining with DAPI. The fluorescent microscopy was visualized with an Olympus FV1000IX81 confocal microscope (Olympus Optical Co. Ltd., Tokyo, Japan). Results are expressed as counted cells in BrdU/NeuN-staining compared to non-irradiated controls as determined by the numbers of cells in randomly selected 5 fields of DG in each slide based on the 8~10 of slides per mouse in experiments conducted in triplicate.

### Immunohistochemistry (IHC)

Expression levels of DCX and MAP-2 in mouse hippocampus were analyzed by IHC as previously described^57^. Frozen mouse brain tissues were fixed in formalin, dehydrated, and embedded in paraffin blocks, which were then sectioned at 4 μm. Sections were incubated in 3% hydrogen peroxide/methanol and then in 0.25% pepsin (S3002; Dako, Carpinteria, CA) to retrieve antigens. They were then blocked in blocking solution (X0909, 10062794; Dako), incubated at 4 °C overnight with primary antibodies diluted in antibody diluent (S3022, 10064048; Dako), washed with TBST, and incubated with polymer-HRP-conjugated secondary antibody (K4001, 10063530 for anti-mouse IgG; K4003, 10061639 for anti-rabbit IgG; Dako). A 3,3′-diaminobenzidine substrate chromogen system (K3468, 10063768; Dako) was used to detect antibody binding. Stained sections were examined under an Olympus IX71 inverted microscope (Olympus Optical Co. Ltd.). The intensity of DAB staining was analyzed using ImageJ/Fiji after color deconvolution for hematoxilin and DAB. Results are expressed as counted cells with Dcx-staining or quantified signals with MAP-2-staining compared to non-irradiated controls as determined by the numbers of cells in the whole area of DG and/or CA regions based on the 8~10 of slides per mouse in experiments conducted in triplicate. Mean DAB intensity within plaques was measured in 10 separate regions (75 × 75 pixel size).

### Cell lines, cell culture, irradiation, and drug treatment

SH-SY5Y and N2a cells purchased from American Type Culture Collection (ATCC, Manassas, VA) were cultured in MEM medium containing 10% fetal bovine serum (FBS), 50 U/mL penicillin, and 50 μg/mL streptomycin (all from Gibco, Paisley, UK), at 37 °C, in humidified air with 5% CO_2_. The medium was changed twice a week and cells were split when they reached approximately 80% confluence. Next, cells were exposed to a single dose of γ-rays using a Gamma Cell-40 Exactor (Nordion International, Inc., Kanata, Ontario, Canada) at a dose rate of 0.81 Gy/min. Plates containing the control cells were placed in the irradiation chamber but not exposed to radiation. The cells were treated with the indicated natural products dissolved in DMSO or distilled water for 24 h.

### Primary cell culture

Pregnant C57BL/6 mice were obtained from a specific pathogen-free colony at Oriental Inc. (Seoul, Korea). Hippocampuses were dissected from postnatal day 2 mice, and prepared for culturing. Following dissection, tissues were chopped into 1 mm^3^ dices, then digested with 10 units/ml papain (Worthington, Freehold, NJ) and 100 units/ml DNase I (Roche, Basel, Switzerland) in DMEM/F-12 at 37 °C for 25 min. The cell suspension was triturated and transferred to T75 culture flasks at a density of 100,000 cells/mL with 10 mL culture medium (DMEM/F-12 with 10% fetal bovine serum, 100 U/mL penicillin, and 100 μg/mL streptomycin) in a 37 °C humidified 5% CO_2_ incubator. The culture medium was changed within 24 h and then twice a week until the cells became confluent. At this time, the flasks were shaken overnight at 200 rpm to remove microglia and oligodendrocytes. Astrocytes were washed three times with PBS and incubated with trypsin/EDTA (0.05% trypsin, 0.53 mM EDTA) for 8 min at 37 °C. The detached cells were then replated at a ratio of 1:2 onto 60 mm plates and grown to 80% confluence prior to irradiation.

### Preparation of conditioned media

Conditioned media from SH-SY5Y and N2a cells were prepared following the previous study^58^. Cells were plated at a density of 5 × 10^4^ cells/mL in 100 mm culture dishes, incubated for 24 h, and then exposed to 2 Gy or 5 Gy of IR. At 2.5 days after irradiation, cells were washed with PBS three times, then further incubated in serum-free media without antibiotics for 36 h. CM were collected and centrifuged to remove any residual cells, after which they were filtered through a 0.2 μm syringe filter. Filtered CM was concentrated 10-fold using a Centricon-10 concentrator (Millipore, Billerica, MA) at 4 °C, then stored at −20 °C. Following CM collection, the number of cells on the dish was determined and the volume of CM used in each experiment was normalized for cell number.

### mRNA extraction and RT-PCR

The expression level of TTR in primary astrocyte was assessed through RT-PCR, as previously described^59^. Total RNA was isolated from irradiated and non-irradiated mice brain samples by Trizol extraction. RNA quality was assessed by agarose gel electrophoresis (visual absence of significant 28S and 18S rRNA degradation) and by spectrophotometry. Total RNA was subjected to RT using SuperScript® VILO cDNA synthesis kit and master mix (Invitrogen, Carlsbad, CA) to obtain cDNAs. RT-PCR was performed using Taq DNA polymerase (Beamsbio, Sungnam, Republic of Korea) and the specific primer pairs (Table S1).

### Real-time quantitative RT-PCR (qRT-PCR)

The expression levels of TTR in SH-SY5Y and N2a cells were assessed through qRT-PCR, as previously described^60^. Aliquots of a master mix containing all of the reaction components with the primers (Table S1) were dispensed into a real time PCR plate (Applied Biosystems, Foster City, CA). All of the PCR reagents were from a SYBR Green core reagent kit (Applied Biosystems). The expression of all genes evaluated was measured in triplicate in the reaction plate. qRT-PCR was performed using an Applied Biosystems-7900 HT qRT-PCR instrument. PCR was performed by subjecting the samples to 15 s at 95 °C and 1 min at 60 °C for 40 cycles followed by thermal denaturation. The expression of each gene relative to *GAPDH* mRNA was determined using the 2^−ΔΔCT^ method. To simplify the data, values for the relative expression were multiplied by 10^2^.

### Gene expression profiling and data analysis

Global gene expression analysis was performed using Affymetrix GeneChip® Human Gene 2.0 ST Arrays, as previously described^61^. RNA qualities were assessed using an Agilent 2100 bioanalyzer and the RNA 6000 Nano Chip (Agilent Technologies), and RNA quantities were determined using a Nanodrop-1000 Spectrophotometer (Thermo Scientific, Hudson, NH). Briefly, 300 ng of total RNA per sample was converted to double-strand cDNA using the procedure recommended by Affymetrix (http://www.affymetrix.com). Using a random hexamer incorporating a T7 promoter, amplified RNA (cRNA) was generated from the double-stranded cDNA template by IVT (*in-vitro* transcription) and then purified using the Affymetrix sample cleanup module. cDNA was regenerated by random-primed reverse transcription using a dNTP mix containing dUTP, and then fragmented using UDG and APE 1 restriction endonucleases and end-labeled with biotinylated dideoxynucleotide using the terminal transferase reaction. Fragmented end-labeled cDNA was hybridized to GeneChip® Human Gene 2.0 ST arrays for 17 h at 45 °C and 60 rpm as described in the Gene Chip Whole Transcript (WT) Sense Target Labeling Assay Manual (Affymetrix). After hybridization, chips were stained and washed in a Genechip Fluidics Station 450 (Affymetrix) and scanned using a Genechip Array scanner 3000 7G (Affymetrix). Expression intensities were extracted from scanned images using Affymetrix Command Console software version 1.1 and stored as CEL files.

Intensity values were normalized to remove bias using the Robust Multi-array Average (RMA) algorithm implemented in the Affymetrix Expression Console software (version 1.3.1.) (http://www.affymetrix.com). Normalized data were exported to programming environment R (version 3.0.2) and overall signal distributions of arrays were compared using plotting using tools obtained from the Bioconductor Project (http://www.bioconductor.org) to confirm normalization. Differentially expressed genes (DEGs) exhibited >1.5-fold average signal differences between control and treatment groups were selected. In addition, the normalized data of selected DEGs were imported into the programming environment R and analyzed using the student’s t-test. Genes found to be differentially expressed with *p*-values of less than 0.05 were subjected to further study. In order to classify gene groups exhibiting similar differential expression patterns, hierarchical clustering analysis was performed using MEV (Multi Experiment Viewer) software version 4.4 (http://www.tm4.org). Finally, the web-based tool DAVID (Database for Annotation, Visualization, and Integrated Discovery) was used to functionally annotate DEGs, which were then classified using gene functional information in the OMIMDISEASE, GENE ONTOLOGY, KEGG PATHWAY, and BIOCARTA databases to identify regulatory networks (http://david.abcc.ncifcrf.gov). Microarray results have been deposited in the Gene Expression Omnibus database (GEO Series accession number GSE94440).

### Western blot analysis

The assessment of protein levels was performed following previous study^62^. Whole cell lysates (WCL) were prepared using radioimmunoprecipitation assay (RIPA) lysis buffer (50 mM Tris, pH 7.4, 150 mM NaCl, 1% Triton X-100, 25 mM NaF, 1 mM dithiothreitol (DTT), 20 mM EGTA, 1 mM Na3VO4, 0.3 mM phenylmethanesulfonyl fluoride (PMSF), and 5 U/mL aprotinin) and the protein concentrations in the lysates were determined using a BioRad protein assay kit (BioRad Laboratories, Hercules, CA). Protein samples were subjected to SDS-PAGE, transferred to a nitrocellulose membrane and then blocked with 5% bovine serum albumin in TBST (10 mM Tris, 100 mM NaCl, and 0.1% Tween 20) for 1 h at room temperature. Next, membranes were probed with specific primary antibodies and peroxidase-conjugated secondary antibody (Santa Cruz Biotechnology). The samples were subsequently analyzed using an ECL detection system (Roche Applied Science, Indianapolis, IN). Densitometric analysis was conducted using the Scion Image software (Scion Corporation, Frederick, MD).

### Enzyme-linked immunosorbent assay (ELISA)

Measurement of retinol in conditioned media was conducted as previously described^63^. Briefly, cells (6 × 10^5^) were plated in 6-well plates and grown to 80% confluence. Following the desired treatments, the concentration of remaining retinol in the media was measured using an enzyme-linked immunoassay kit (Gentaur, Kampenhout, Belgium), according to the manufacturer’s instructions.

### Statistical analysis

All numeric data are presented as the means ± standard deviation (SD) from at least three independent experiments. Experimental results were analyzed by one-way ANOVA for ranked data followed by Tukey’s honestly significant difference test. The Prism 5 software (GraphPad Software, SanDiego, CA) was used to conduct all statistical analyses. A *p*-value < 0.05 was considered to be statistically significant.

## Electronic supplementary material

Supplementary Information
