# Supplementary material for: Radiation-induced overexpression of transthyretin inhibits retinol-mediated hippocampal neurogenesis
Source: Sci Rep. 2018 May 30;8:8394. doi: 10.1038/s41598-018-26762-1 (PMC5976673; doi:10.1038/s41598-018-26762-1)
Supplement: Supplementary file 1 — Supplementary Information [file 41598_2018_26762_MOESM1_ESM.doc]

**Radiation-induced overexpression of transthyretin inhibits retinol-mediated hippocampal neurogenesis**

**JiHoon Kang^a,1^, Wanyeon Kim^b,1,2^, HyunJeong Seo^a^, EunGi Kim^a^, Beomseok Son^a^, Sungmin Lee^a^, Gaeul Park^a^, Sunmi Jo^c^, Changjong Moon^d^, HyeSook Youn^e,*^, and BuHyun Youn^a,b,*^**

^a^Department of Integrated Biological Science, Pusan National University, Busan 46241, Republic of Korea; ^b^Department of Biological Sciences, Pusan National University, Busan 46241, Republic of Korea; ^c^Department of Radiation Oncology, Haeundae Paik Hospital, Inje University School of Medicine, Busan 48108, Republic of Korea; ^d^Department of Veterinary Anatomy, College of Veterinary Medicine and Animal Medical Institute, Chonnam National University, Gwangju 61186, Republic of Korea; ^e^Department of Integrative Bioscience and Biotechnology, Sejong University, Seoul 05006, Republic of Korea

^1^These authors contributed equally to the work.

^2^Present address: Department of Biology Education, Korea National University of Education, Cheongju 28173, Republic of Korea.

^*^Corresponding to:
**BuHyun Youn**, Department of Biological Sciences, Pusan National University, Busandaehak-ro 63beon-gil, Geumjeong-gu, Busan 46241, Republic of Korea.
Tel: 82-51-510-2264; Fax: 82-51-581-2962; e-mail: bhyoun72@pusan.ac.kr

**HyeSook Youn**, Department of Integrative Bioscience and Biotechnology, Sejong University, 209 Neungdong-ro, Gwangjin-gu, Seoul 05006, Republic of Korea.
Tel: 82-2-6935-2438; Fax: 82-2-3408-4334; e-mail: hsyoun@sejong.ac.kr

**
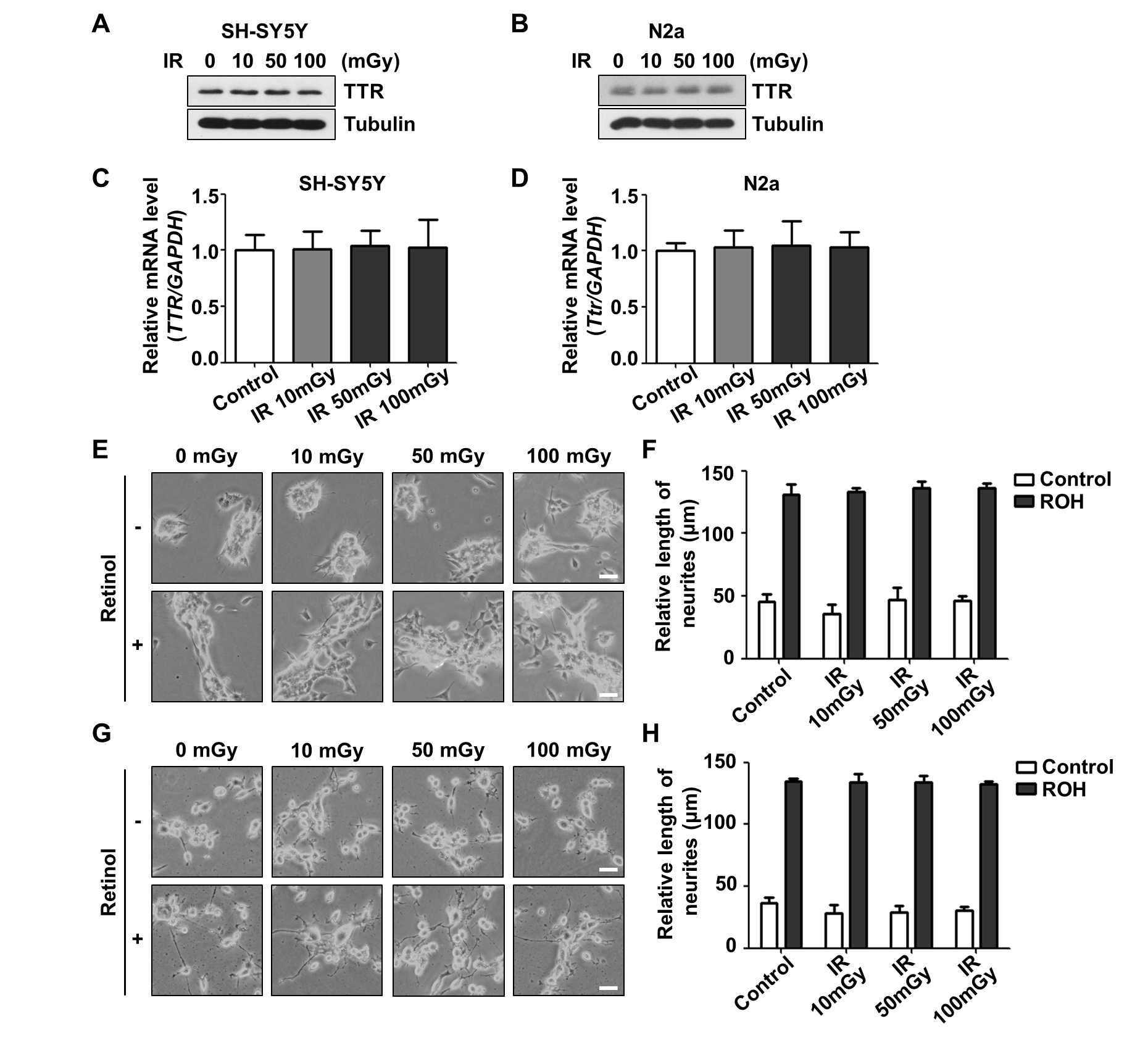
**

**Supplementary Figure S1**. Exposure to low-dose radiation increased TTR expression *in vitro*. (A and B) The expression levels of TTR after treatment of low-dose radiation in SY-SY5Y and N2a cells were quantified by western blot analysis. Both cells were treated with 0, 10, 50, 100 mGy of radiation and incubated for 24 h. Tubulin was used for equal quantification. Full-length blots are presented in Fig. S7. (C and D) *TTR* mRNA levels in SH-SY5Y and N2a cells in response to low-dose radiation were analyzed by qRT-PCR and normalized using *GAPDH* mRNA as the internal control. (E) The effects of low-dose radiation on retinol-mediated neuritogenesis in SH-SY5Y cells were investigated by microscopic observation. Scale bar is 100 μm. (F) The effects of low-dose radiation on retinol-mediated neuritogenesis in SH-SY5Y cells were quantified and analyzed. (G) The effects of low-dose radiation on retinol-mediated neuritogenesis in SH-SY5Y cells were investigated by microscopic observation. (H) The effects of low-dose radiation on retinol-mediated neuritogenesis in N2a cells were quantified and analyzed.

**
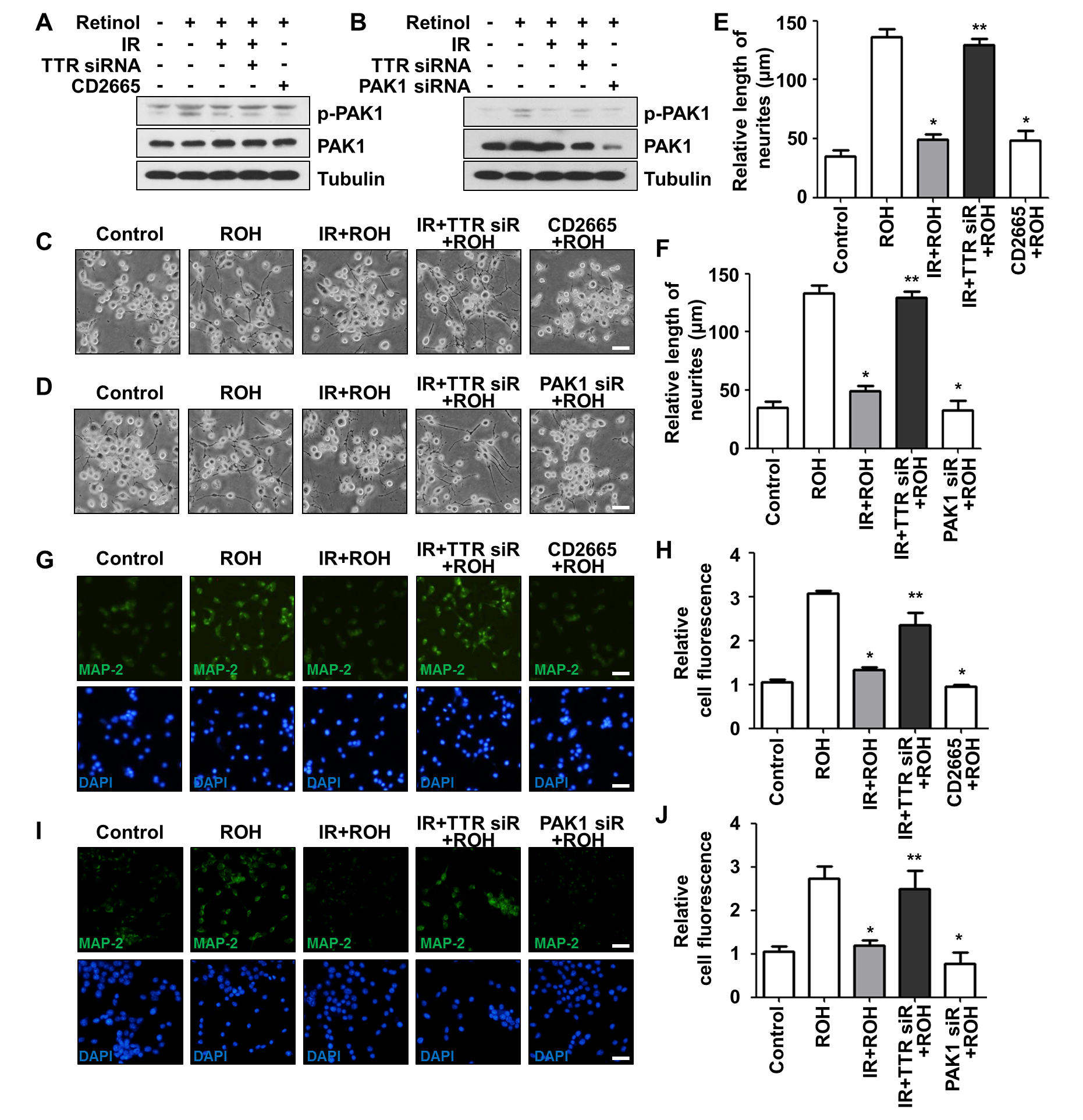
**

**Supplementary Figure S2**. Neuritogenesis after retinol uptake was mediated by the RAR-Rac1-PAK1 signaling axis. (A) The inhibitory effects of TTR on retinol-mediated PAK1 phosphorylation in N2a cells were investigated by Western blot analysis. N2a cells were transfected with control siRNA or TTR siRNA for 48 h, then irradiated or treated with CD2665. At 24 h after retinol treatment, cells were trypsinized and lysates were used for analysis. Tubulin was used as an internal control. Full-length blots are presented in Fig. S7. (B) The effects of TTR on retinol-mediated PAK1 phosphorylation in N2a cells were confirmed using PAK1-specific siRNA. Full-length blots are presented in Fig. S7. (C) The effects of TTR-mediated PAK1 inhibition on neurite outgrowth were investigated by microscopic observation. Scale bar is 100 μm. (D) The effects of TTR-mediated PAK1 inhibition on neurite outgrowth were confirmed by knockdown of PAK1 using specific siRNA. (E) The effects of TTR-mediated PAK1 inhibition on neurite outgrowth were quantified by evaluating the length of neurites and statistically analyzed. * *p* < 0.05 vs. retinol-treated cells. ** *p* < 0.05 vs. irradiated and retinol-treated cells. (F) The effects of TTR-mediated PAK1 inhibition on neurite outgrowth were quantified by measuring the length of neurites and statistically analyzed. * *p* < 0.05 vs. retinol-treated cells. ** *p* < 0.05 vs. irradiated and retinol-treated cells. (G) The effects of TTR-mediated PAK1 inhibition on MAP-2 expression were investigated by immunocytochemistry. (H) The effects of TTR-mediated PAK1 inhibition on MAP-2 expression were quantified and analyzed by ImageJ/Fiji program. * *p* < 0.05 vs. retinol-treated cells. ** *p* < 0.05 vs. irradiated and retinol-treated cells. (I) The effects of TTR-mediated PAK1 inhibition on MAP-2 expression were confirmed by using PAK1-specific siRNA. (J) The effects of TTR-mediated PAK1 inhibition on MAP-2 expression were quantified and analyzed. * *p* < 0.05 vs. retinol-treated cells. ** *p* < 0.05 vs. irradiated and retinol-treated cells.


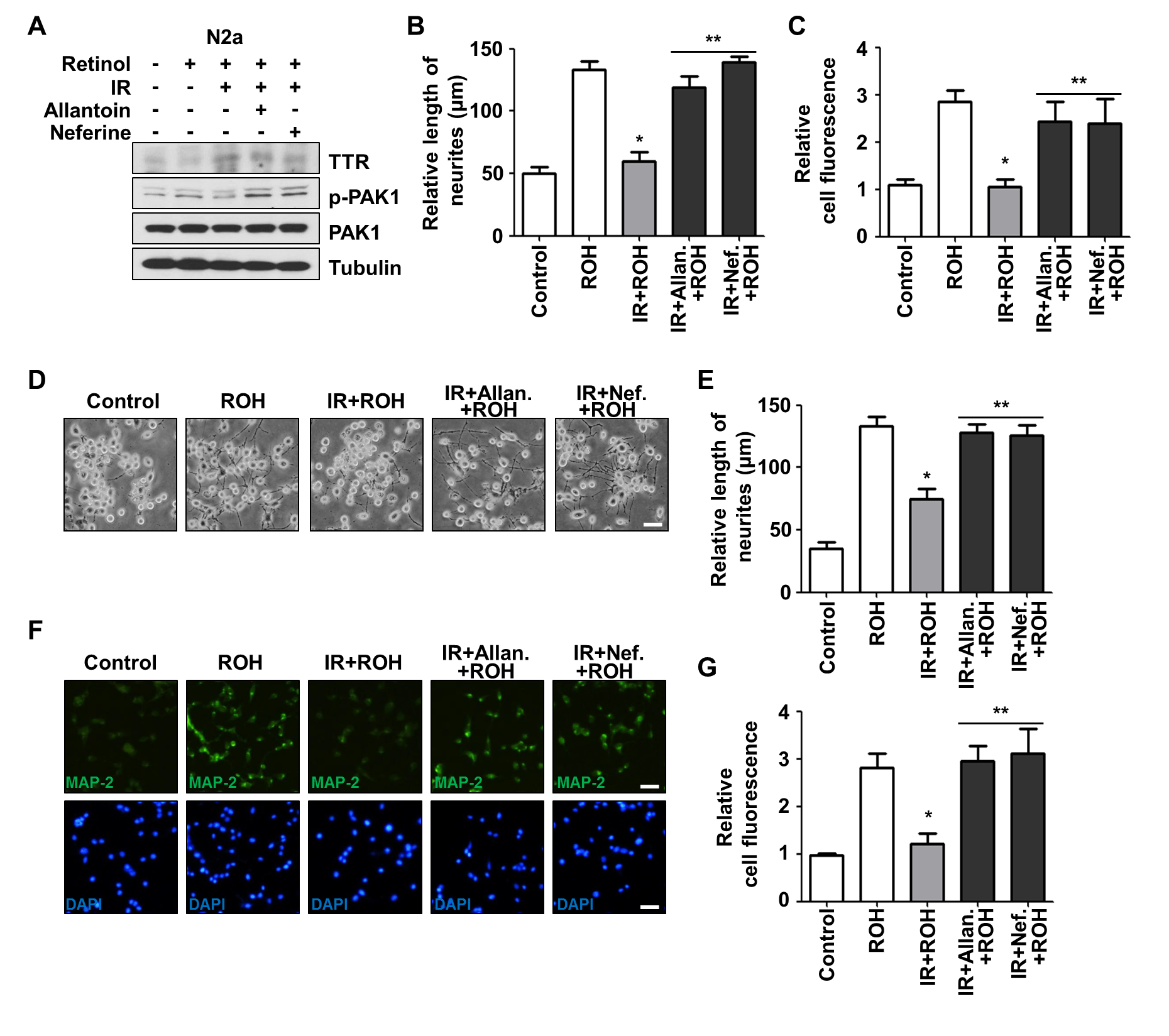


**Supplementary Figure S3**. Allantoin and neferine rescued IR-induced decrease of neurogenesis and depression-like symptoms. (A) The effects of both allantoin and neferine on TTR expression and PAK1 phosphorylation in N2a cells were analyzed by western blot assay. N2a cells were treated with 10 μM of allantoin or 2 μM of neferine with irradiation and incubated for 24 h. After 24 h following treatment of retinol, cells were trypsinized and lysates were used for analysis. Tubulin was utilized for equal quantification. Full-length blots are presented in Fig. S7. (B) The effects of TTR inhibition by allantoin and neferine on neurite outgrowth in SH-SY5Y cells were quantified and analyzed (corresponding to Fig. 5B). * *p* < 0.05 vs. retinol-treated cells. ** *p* < 0.05 vs. irradiated and retinol-treated cells. (C) The effects of TTR inhibition by allantoin and neferine on MAP-2 expression in SH-SY5Y cells were quantified and analyzed (corresponding to Fig. 5C). * *p* < 0.05 vs. retinol-treated cells. ** *p* < 0.05 vs. irradiated and retinol-treated cells. (D) The effects of TTR inhibition by allantoin and neferine on IR-mediated repression of neurite outgrowth were analyzed by microscopic observation. Scale bar is 100 μm. (E) The effects of TTR inhibition by allantoin and neferine on neurite outgrowth in N2a cells were quantified and analyzed. * *p* < 0.05 vs. retinol-treated cells. ** *p* < 0.05 vs. irradiated and retinol-treated cells. (F) The effects of TTR inhibition by allantoin and neferine on IR-mediated repression MAP-2 expression were investigated by immunocytochemistry. (G) The effects of TTR inhibition by allantoin and neferine on MAP-2 expression in N2a cells were quantified and analyzed. * *p* < 0.05 vs. retinol-treated cells. ** *p* < 0.05 vs. irradiated and retinol-treated cells.


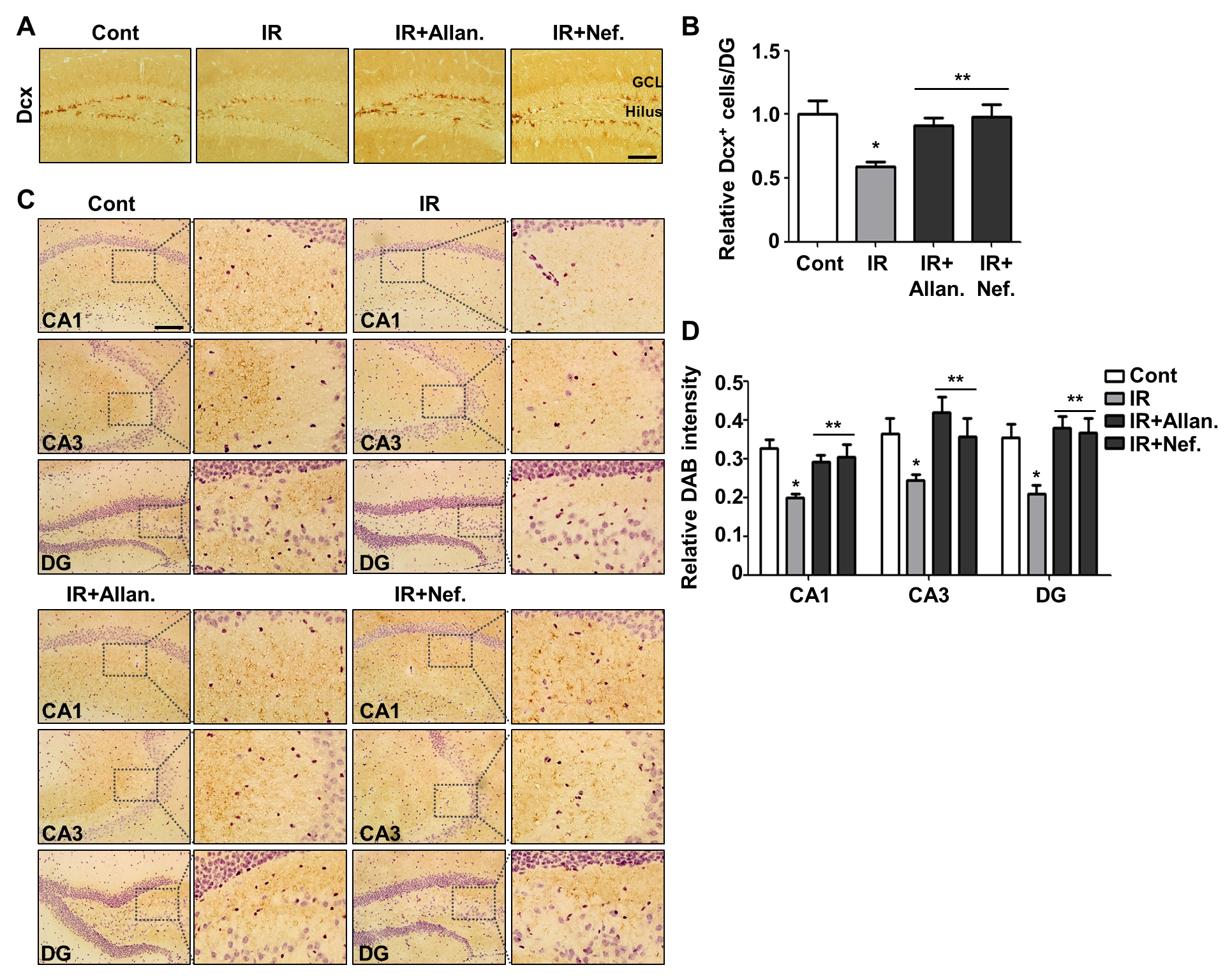


**Supplementary Figure S4**. Allantoin and neferine rescued IR-induced decrease of neurogenesis *in vivo* model. (A) The effects of allantoin and neferine on doublecortin (Dcx) expression in hippocampal regions of irradiated mice were analyzed by IHC (GCL-Granule Cell Layer). C57BL/6 mice were treated with cranial radiation and injected intraperitoneally with 30 mg/kg of allantoin and 15 mg/kg of neferine per 3 days. Scale bar is 50 μm. (B) The Dcx-stained cells in IHC counted in the whole area of DG and statistically analyzed. * *p* < 0.05 vs. control mice. (C) The effects of allantoin and neferine on MAP-2 expression in hippocampal regions of cranially irradiated mouse were analyzed by IHC. (CA1, *Cornu Ammonis* 1; CA3, *Cornu Ammonis* 3; DG, dentate gyrus). The magnification of images was ×200 (left) and ×400 (right), respectively. (D) The MAP-2 staining intensity in IHC was quantified and analyzed using ImageJ/Fiji program. * *p* < 0.05 vs. control mice. ** *p* < 0.05 vs. irradiated mice.


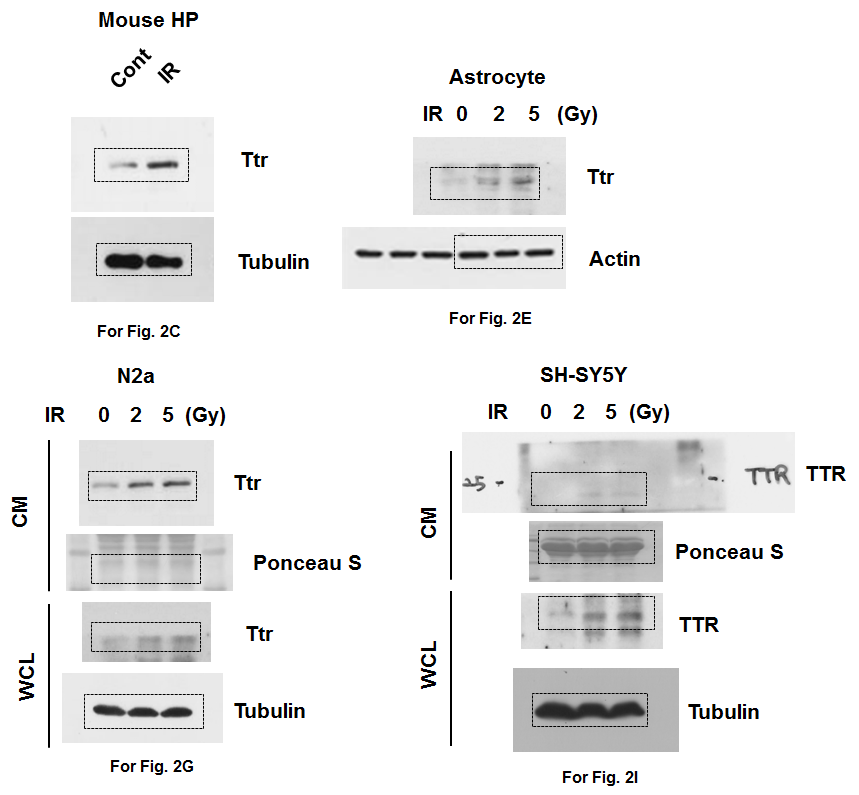


**Supplementary Figure S5**. Full-length blots for Fig. 2C, 2E, 2G, and 2I

**
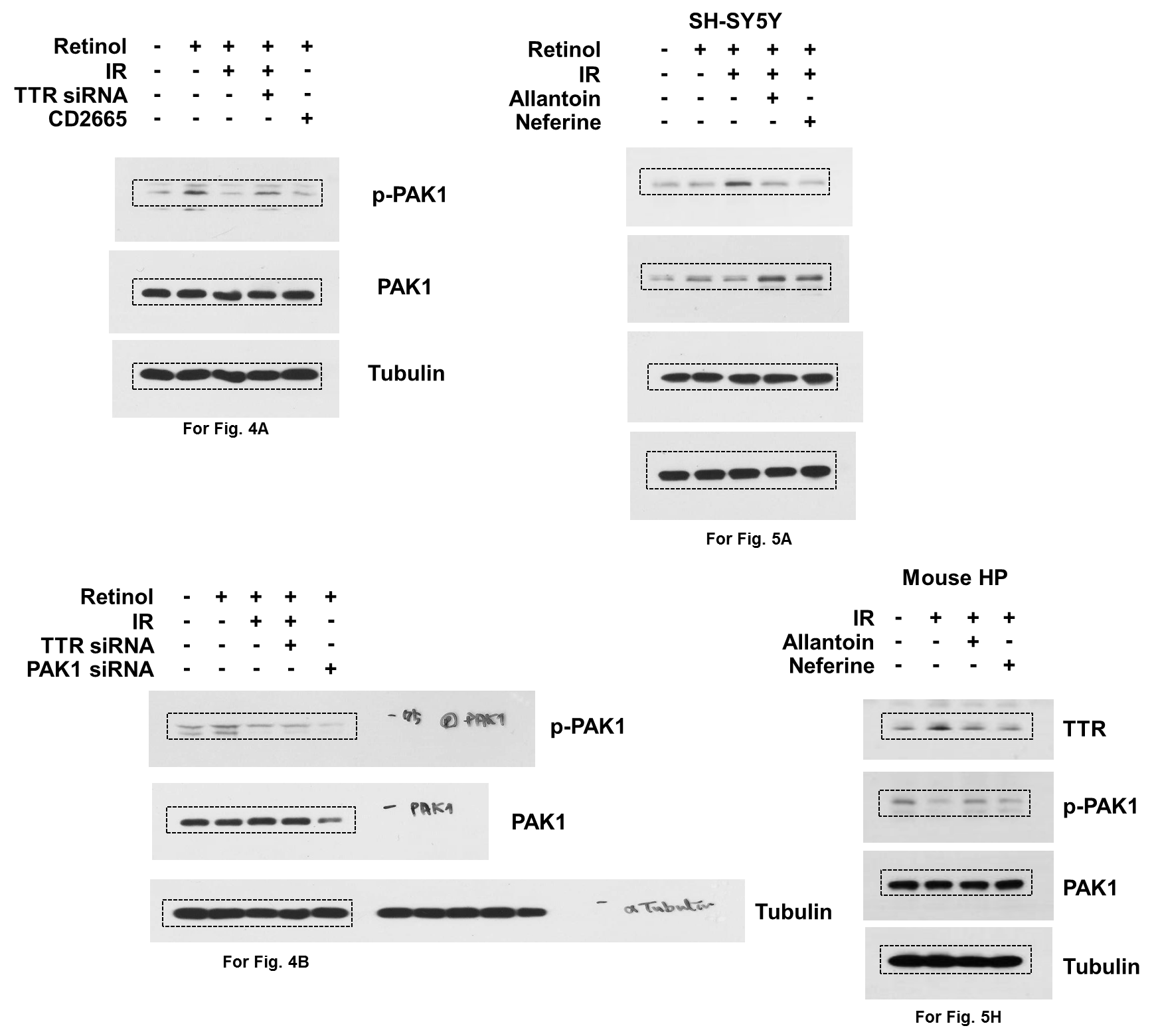
**

**Supplementary Figure S6**. Full-length blots for Fig. 4A, 4B, 5A, and 5H

**
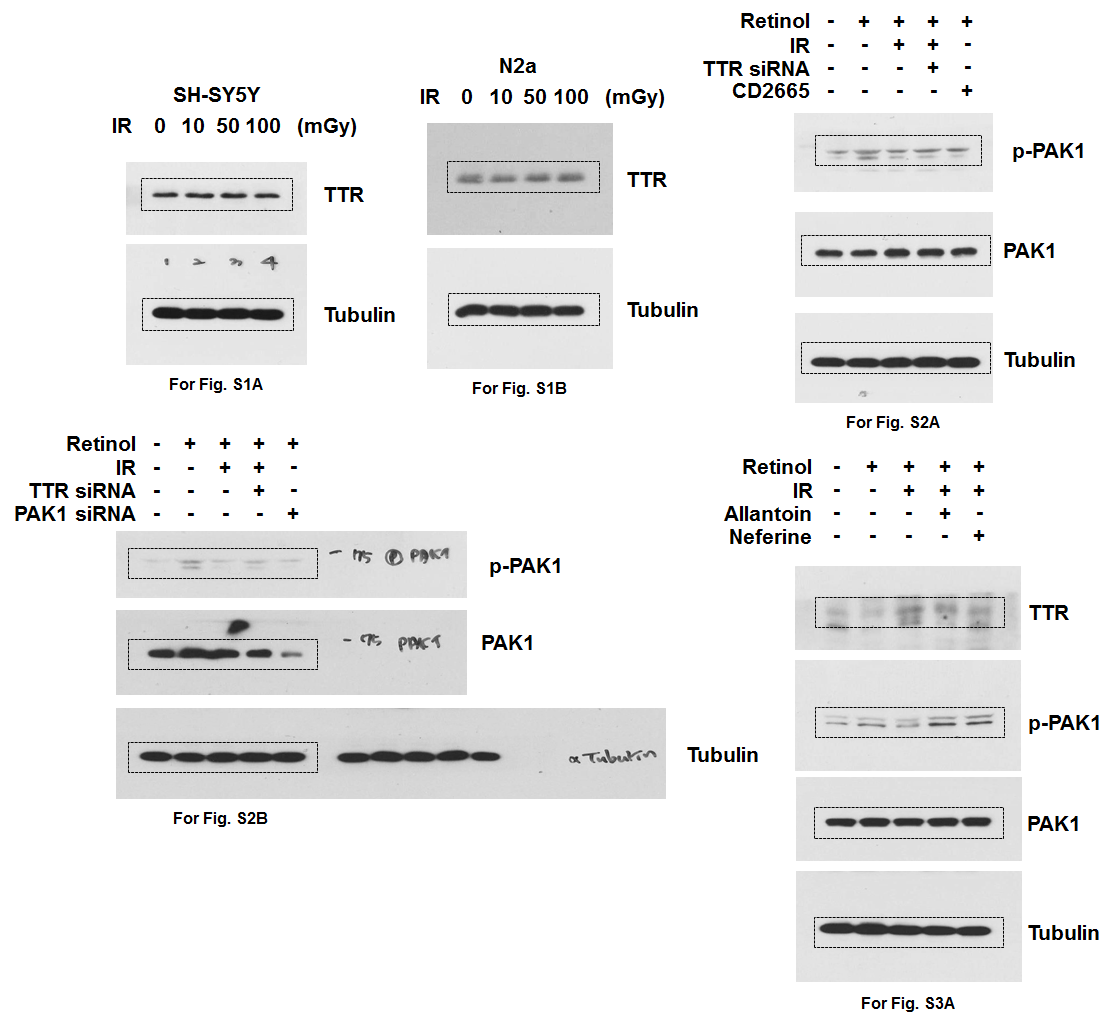
**

**Supplementary Figure S7**. Full-length blots for Fig. S1A, S1B, S2A, S2B, and S3A

**Table S1.** Primers for determining expression levels of TTR

| Gene name | Forward primer | Reverse primer |
| --- | --- | --- |
| *Ttr* (mouse) | 5’-AGCCCTTTGCCTCTGGGAAGA-3’ | 5’-TGCGATGGTGTAGTGGCGATGG-3’ |
| *18S* (mouse) | 5’-CTGGATACCGCAGCTAGGAA-3’ | 5’-CCCTCTTAATCATGGCCTCA-3’’ |
| *TTR* (human) | 5’-TTCTAGATGCTGTCCGAGGC-3’ | 5’-TCATGGAATGGGGAGATGCC-3 |
| *GAPDH* (human) | 5′-GCTAGGGACGGCCTGAAG-3′ | 5′-GCCCAATACGACCAAATCC-3′ |
